# Correction: Differential regulation of BK channels by fragile X mental retardation protein

**DOI:** 10.1085/jgp.20191250205042020c

**Published:** 2020-05-22

**Authors:** Aravind Kshatri, Alejandro Cerrada, Roger Gimeno, David Bartolomé-Martín, Patricio Rojas, Teresa Giraldez

Vol. 152, No. 6 | 10.1085/jgp.201912502 | April 10, 2020

*JGP* regrets that in the original version of this paper, an error was made in Table 1. In the BKαβ_4_ column, the value for *L*_0_ has been corrected and is shown in bold in the table below.

Both the HTML and PDF versions of the article have been corrected. This error appears only in PDF versions downloaded on or before May 6, 2020.

**Table 1.** Best fit parameters for P_o_-V data with the HA model

Parameter	BKα	+FMRP	BKαβ_4_	+FMRP
*L*_0_	3.6 × 10^−6^	6.9 × 10^−6^	**9.8 × 10**^**−7**^	8.6 × 10^−6^
*Z*_*L*_	0.26	0.29	0.23	0.27
*J*_0_	0.05	0.17	0.12	0.15
*Z*_*J*_	0.61	0.58	0.59	0.55
*D*	13	13	16	11
*V*_*HC, mV*_	124	77	91	87
*V*_*HO, mV*_	18	−35	−28	−23

